# Establishment and Application of Multiplex PCR for Simultaneously Detecting *Escherichia coli, Salmonella, Klebsiella pneumoniae*, and *Staphylococcus aureus* in Minks

**DOI:** 10.3389/fvets.2020.588173

**Published:** 2020-11-17

**Authors:** Peng Li, Dingxiu Zhang, Hongmei Li, Jinying Pang, Huijun Guo, Jianhua Qiu

**Affiliations:** ^1^College of Animal Science and Veterinary Medicine, Shandong Agricultural University, Tai'an, China; ^2^Shandong Provincial Key Laboratory of Animal Biotechnology and Disease Control and Prevention, Shandong Provincial Engineering Technology Research Center of Animal Disease Control and Prevention, Shandong Agricultural University, Tai'an, China

**Keywords:** minks, *E. coli*, *Salmonella*, *K. pneumoniae*, *S. aureus*, multiplex PCR detection

## Abstract

To establish a multiplex PCR for simultaneous detection of *Escherichia coli* (*E. coli*), *Salmonella, Klebsiella pneumoniae* (*K. pneumoniae*), and *Staphylococcus aureus* (*S. aureus*), four pairs of specific primers were designed according to the conservative regions of *phoA* gene for *E. coli, invA* gene for *Salmonella, khe* gene for *K. pneumoniae, nuc* gene for *S. aureus*. The quadruple PCR system was established through optimization of multiplex PCR and detection of specificity, sensitivity, and stability. The results showed that target gene bands of *E. coli* (622 bp), *Salmonella* (801 bp), *K. pneumoniae* (303 bp), and *S. aureus* (464 bp) could be amplified by this method specifically and simultaneously from the same sample containing the four pathogens, with a detection sensitivity of 100 pg/μL. Meanwhile, no bands of common clinical bacteria, including *Clostridium perfringens, Pseudomonas aeruginosa, Pasteurella multocida, Streptococcus pneumoniae, Streptococcus pneumoniae, Proteus mirabilis, Staphylococcus sciuri, Staphylococcus pseudintermedius, Acinetobacter baumannii, Enterococcus faecalis*, and *Bacillus subtilis* were amplified. In addition, 380 tissue samples were detected by multiplex and single PCR established in current study, respectively. Among the 368 carcass samples, positive detection rates of *E. coli, K. pneumoniae, Salmonella*, and *S. aureus* were 33.7, 12.0, 10.6, and 13.9%. Among the 12 visceral tissue samples, positive detection rates of *E. coli, K. pneumoniae, Salmonella*, and *S. aureus* were 41.7, 25.0, 16.7, and 8.3%, respectively. Positive detection rates of multiplex PCR were consistent with that of single PCR. Compared with single PCR, the multiplex PCR method had the advantages of time-saving, high specificity and high sensitivity. The results showed that the minks in these farms had mixed infection of these four pathogens, and the method established in this study could be applied to the rapid and accurate detection and identification of these four bacteria. In conclusion, the multiplex PCR method has stable detection results, good repeatability, and short detection time. It is suitable for the rapid and accurate detection of four kinds of bacteria above the carcass of fur animals, which could be suitable in microbial epidemiology investigation. It can provide a reliable technical reference for rapid clinical diagnosis and detection.

## Introduction

As a valuable economic fur animal, the domestic breeding of minks has been paid more and more attention. The scale of mink breeding in the world in 2014 is 100 million, while that in China is close to 80 million (1). And Shandong province is the largest province for mink breeding and skin production. The mink skins production in Shandong corresponded to 70% of all skins produced in China in 2016 (2). Although in recent years, due to the influence of foreign market demand, the breeding scale has declined, but mink breeding is still an important pillar industry in Shandong Province. However, a range of pathogenic microbes are causing a wide variety of infectious diseases in Shandong (3–8). Some of which may even have the risk of infecting people, and it would be a threat to mink breeding and public health. At the same time, bacterial disease is one of the main causes of mink death.

*Escherichia coli* (*E. coli*), *Salmonella, Klebsiella pneumoniae* (*K. pneumoniae*), and *Staphylococcus aureus* (*S. aureus*) are the most common pathogens or opportunistic pathogens in minks. They can infect not only minks, but also human, and other animals (9–12). In recent years bacterial diseases have been not only frequent, but also in the state of mixed infection or secondary infection in mink farms of Shandong (13, 14). Therefore, it is necessary to give timely and rapid diagnosis and treatment of multiple infection in mink culture.

The traditional method of bacterial pathogens identification mostly uses bacterial isolation and culture combined with biochemical characteristics detection and analysis. Although the traditional methods are reliable, there are still some shortcomings that affect their application, such as strong specialization and high requirements for operators, high risk, time-consuming, and expensive.

Modern molecular biology technology, such as polymerase chain reaction (PCR) combined with gel electrophoresis, is increasingly used in bacteria isolation and identification, which improves the detection efficiency. Compared with single PCR, multiplex PCR has higher detection efficiency, which can not only detect a variety of pathogens at the same time, but also reduce the cost and save the time. It can be seen that multiplex PCR detection is of great significance for the rapid diagnosis and detection of mixed infection (15–17). However, the research on the simultaneous detection of *E. coli, Salmonella, K. Pneumoniae*, and *S. aureus* in minks by quadruple PCR in Shandong has not been reported, and there is still a lack of relevant research data.

Therefore, the current study was conducted to design primers according to the published gene sequences of these four mink bacteria, and then to establish a multiplex PCR method for simultaneous detection of four bacteria. So as to provide a reference for the rapid identification of these four common or opportunistic pathogenic bacteria.

## Materials and Methods

### Source of Bacterial Strains

Reference strains of *E. coli* (ATCC8739), *Salmonella* (ATCC13076), *K. pneumoniae* (CMCC46117), and *S. aureus* (ATCC6538) used in this study were all purchased from China microbial strain network. *Clostridium perfringens, Pseudomonas aeruginosa, Pasteurella multocida, Streptococcus pneumoniae, Streptococcus pneumoniae, Proteus mirabilis, Staphylococcus sciuri, Staphylococcus pseudintermedius, Acinetobacter baumannii, Enterococcus faecalis*, and *Bacillus subtilis* positive strains were all pathogenic bacteria isolated from minks, which were isolated, identified and preserved by the laboratory of College of Animal Science and Technology, Shandong Agricultural University.

### Sampling and Isolation

During January 2017 to October 2018, 380 samples, including 368 carcass samples (from right hind leg) and 12 visceral tissue samples from diseased minks, were collected from 35 mink farms in the main mink-producing areas of Shandong Province, China, including Zhucheng, Wendeng, Liaocheng, Rizhao, Haiyang, and Dongping. Among the 12 diseased mink tissue samples, seven minks had obvious clinical symptoms of respiratory diseases, and the remaining five minks had clinical symptoms of digestive tract diseases. These samples were used for bacterial isolation by traditional clinical microbilologic methods, and then the established multiplex PCR method and the single PCR method were used to detect these bacterial isolation, and the multiplex PCR results were compared with the single PCR method to detect the specificity of multiplex PCR method.

### Primers

The target genes chosen for their specificity were the *phoA* gene in *E. coli, invA* gene in *Salmonella, khe* gene in *K. pneumoniae* and *nuc* gene in *S. aureus* (6, 15–21). Four pairs of specific primers were designed by Premier 5.0 and Oligo 6.0 along with NCBI primer-BLAST comparison. All the primers used in current study were synthesized by Shanghai Sangon Biotech Co., Ltd. The primers and the respective amplification lengths are shown in Table 1.

**Table 1:** Primers used in multiplex PCR.

**Bacteria**	**Primer sequences (5^**′**^-3^**′**^)**	**Target genes**	**Gene ID**	**PCR product size (bp)**
*K. pneumoniae*	F-CGATGCTACTTATCCCGACA R-ACCACCAGCAGACGAACTT	*khe*	KX842080.1	303
*S. aureus*	F-AGGCATGGCTATCAGTAATGTTTC R-CATCAGCATAAATATACGCTAAGCCAC	*nuc*	DQ507382.1	464
*E. coli*	F-TACAGGTGACTGCGGGCTTATC R-CTTACCGGGCAATACACTCACTA	*phoA*	FJ546461.1	622
*Salmonella*	F-AAAAGAAGGGTCGTCGTTAG R-GGAAGGTACTGCCAGAGGTC	*invA*	MK017941.1	801

### DNA Extraction

The reference strains and the positive isolates identified by traditional clinical microbiologic methods were inoculated on 5 mL Tryptic soy broth (TSB), and then cultured by shaking at 37°C for 18~24 h. Bacterial genomic DNA was extracted from 1 mL bacterial solution with a TIANamp Bacterial DNA Kit (Tiangen, Beijing, China) according to the Kit instructions. The extracted bacterial DNA was detected by nucleic acid analyzer and diluted to 10 ng/μL for subsequent test.

Genomic DNA of carcass samples and diseased mink visceral tissue samples were extracted using TIANamp Blood/Cell/Tissue DNA Kit (Tiangen, Beijing, China) according to the Kit instructions. The extracted DNA was stored at −20°C.

### Control, Optimization, and Establishment of Multiplex PCR Conditions

Firstly, the genomic DNA of four standard positive strains was used as a template to screen the optimal annealing temperature by single PCR reaction. Ten annealing temperature gradients of 52, 52.9, 53.8, 54.9, 56, 57, 58.1, 59.2, 60.1, and 61°C were set to determine the optimal annealing temperature. Next, multiplex PCR was performed using the same volume mixture of genomic DNA of four standard strains as template. Thus, the concentration of primers and annealing temperature were optimized to determine the optimal multiplex PCR reaction conditions.

The results of the preliminary test showed the multiplex PCR reaction were carried out in 25 μL reaction mixtures containing 12.5 μL of 2 × Es Taq MasterMix, 1 μL for each of the four bacterial DNA templates, 1 μL for each of the four pairs of primers with the best concentration ratio. Among which Taq MasterMix was composed of Es Taq DNA Polymerase (amplification efficiency: 2 kb/min), MgCl_2_ (3 mM/L), dNTP(400 μM/L), PCR stabilizer, and enhancer. Finally, the volume of the reaction mixtures was filled up to 25 μL with sterilized double distilled water. The amplification conditions consisted of an initial denaturation at 94°C for 7 min, 30 cycles of denaturation at 94°C for 30 s, annealing at 52 61°C for 30 s, extension at 72°C for 30 s, and final extension for 5 min at 72°C. On the basis of four optimum concentration ratio primers, 10 annealing temperature gradients of 52, 52.9, 53.8, 54.9, 56, 57, 58.1, 59.2, 60.1, and 61°C were also set to select the annealing temperature and optimize the reaction conditions.

### Sensitivity Test of Multiplex PCR

The DNA template was serially diluted to 10 ng/μL, 1 ng/μL, 100 pg/μL, 10 pg/μL, 1 pg/μL, 100 fg/μL by 10 fold gradient with sterile double distilled water, then amplified by the optimized single PCR reaction system. Subsequently, the DNA template was serially diluted to 100 ng/μL, 10 ng/μL, 1 ng/μL, 100 pg/μL, 10 pg/μL, 1 pg/μL by 10 fold gradient with sterile double distilled water, then amplified by the optimized multiplex PCR reaction system. Finally, the PCR products were electrophoresis to detect the sensitivity of each primer.

### Specificity Test of Multiplex PCR

The mixed DNA or single DNA of four standard strains and the DNA of common bacteria samples in clinic, such as *Clostridium perfringens, Pasteurella multocida, Pseudomonas aeruginosa, Streptococcus pneumoniae, Streptococcus pneumoniae, Proteus mirabilis, Staphylococcus sciuri, Staphylococcus pseudintermedius, Acinetobacter baumannii, Enterococcus faecalis*, and *Bacillus subtilis* were used as templates in the optimized reaction system for multiplex PCR amplification to detect the specificity of primers.

### Stability Test of the Multiplex PCR

In order to evaluate the stability of the multiplex PCR system, templates of the positive and negative samples, respectively, or mixed were added into reaction mixture, and then were amplified by the optimized multiplex PCR system. The positive control was four bacteria DNA (*E. coli, Salmonella, K. Pneumoniae*, and *S. aureus*), which were identified as positive by biochemical detection and 16S rRNA sequencing. The negative control was sterilized double distilled water. The stability test was repeated three times. In addition, the detection effect of multiplex PCR system was evaluated by comparing with single PCR.

## Result

### Establishment of Multiplex PCR Conditions

**Figure 1:**
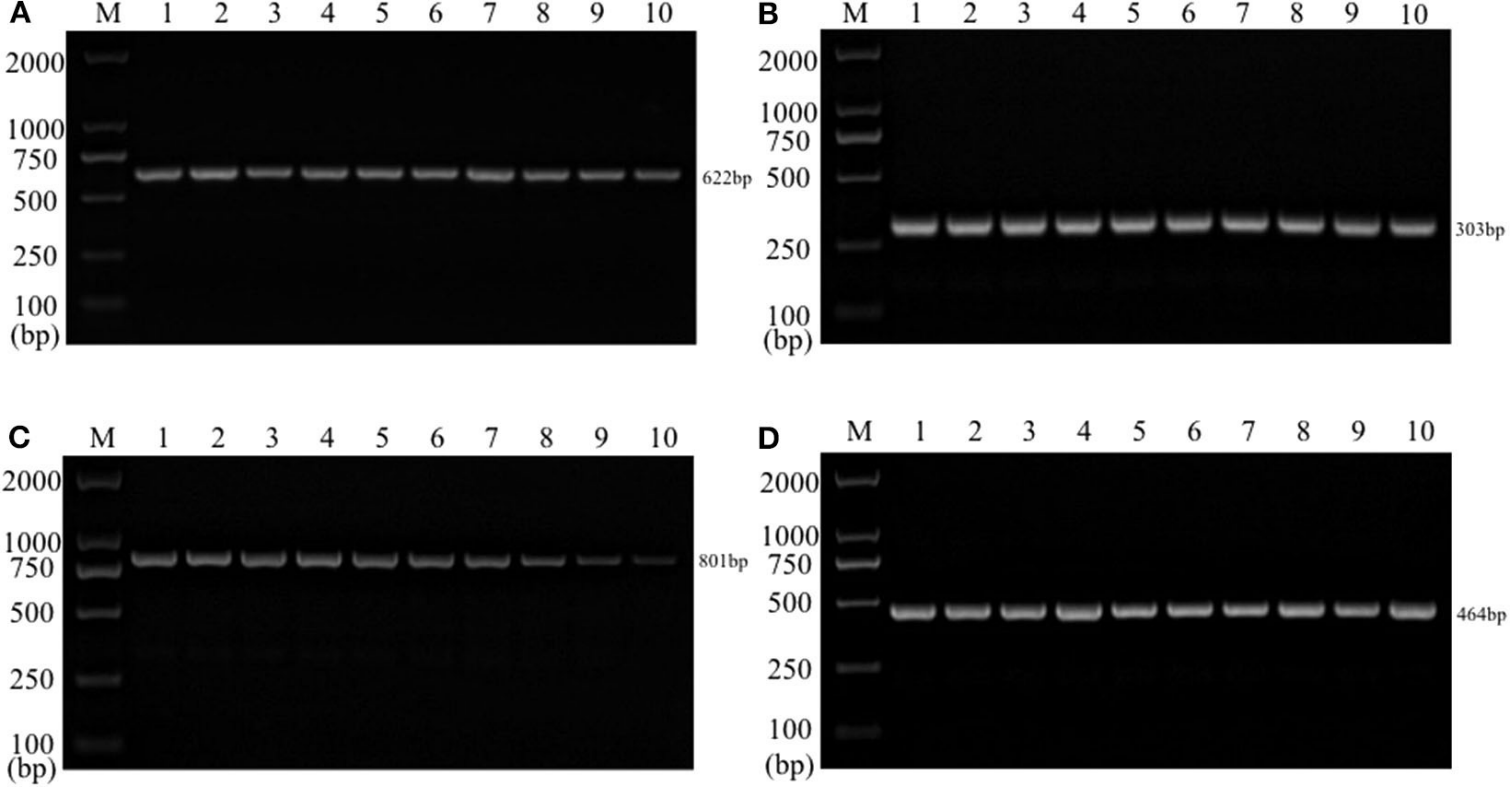
Single PCR amplification at different annealing temperature. Lane M, D2000 DNA marker; Lane 1, 52.0°C; Lane 2, 52.9°C; Lane 3, 53.8°C; Lane 4, 54.9°C; Lane 5, 56.0°C; Lane 6, 57.0°C; Lane 7, 58.1°C; Lane 8, 59.2°C; Lane 9, 60.1°C; Lane 10, 61.0°C. **(A)** *E. coli*; **(B)** *K. pneumoniae*; **(C)** *Salmonella*; **(D)** *S. aureus*.

**Figure 2:**
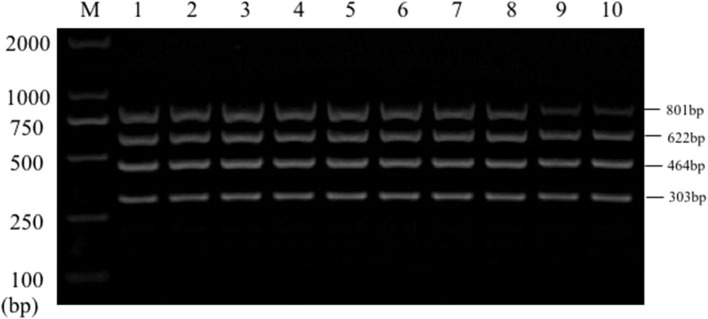
Multiplex PCR amplification at different annealing temperature. Lane M, D2000 DNA marker; Lane 1, 52.0°C; Lane 2, 52.9°C; Lane 3, 53.8°C; Lane 4, 54.9°C; Lane 5, 56.0°C; Lane 6, 57.0°C; Lane 7, 58.1°C; Lane 8, 59.2°C; Lane 9, 60.1°C; Lane 10, 61.0°C.

Single PCR test results of the each reference bacteria DNA showed that, the specific gene amplification products of the four bacteria DNA were obtained, which were 622 bp for *E. coli*, 801 bp for *Salmonella*, 303 bp for *K. Pneumoniae*, and 464 bp for *S. aureus*, respectively. The results of multiplex PCR combined with single PCR showed that when the annealing temperature was 56°C, the amplified bands of four target genes of *E. coli, Salmonella, K. Pneumoniae*, and *S. aureus* were uniform, concentrated and highly specific (Figures 1, 2). The subsequent tests were carried out at 56°C.

### Results of Multiplex PCR Sensitivity Experiment

**Figure 3:**
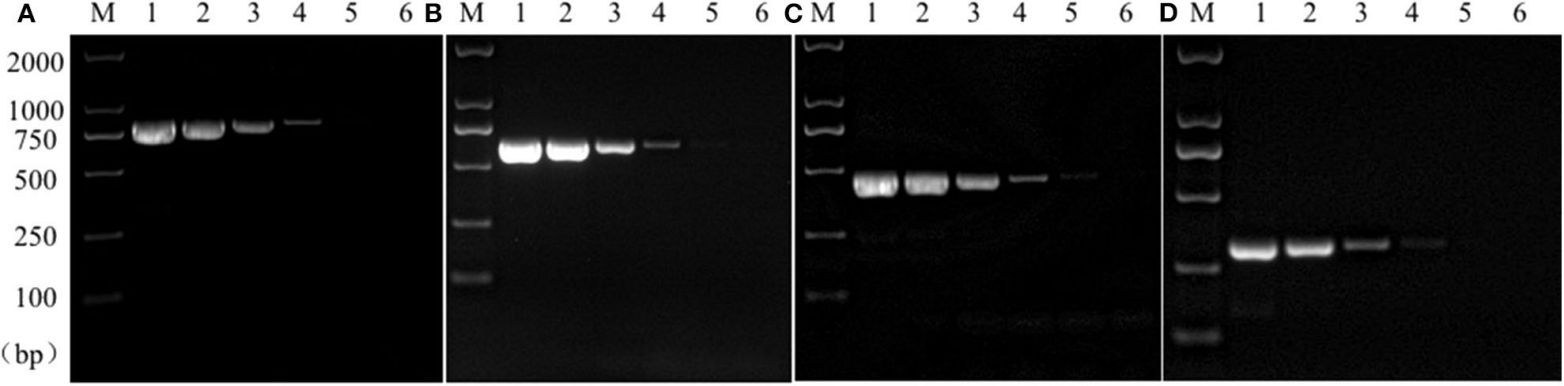
Results of single PCR assay sensitivity experiment. Lane M, D2000 DNA marker; Lane 1~6: The concentration was 10 ng/μL, 1 ng/μL, 100 pg/μL, 10 pg/μL, 1 pg/μL, and 100 fg/μL, respectively; **(A)** *Salmonella*; **(B)** *E. coli*; **(C)** *S. aureus*; **(D)** *K. pneumoniae*.

**Figure 4:**
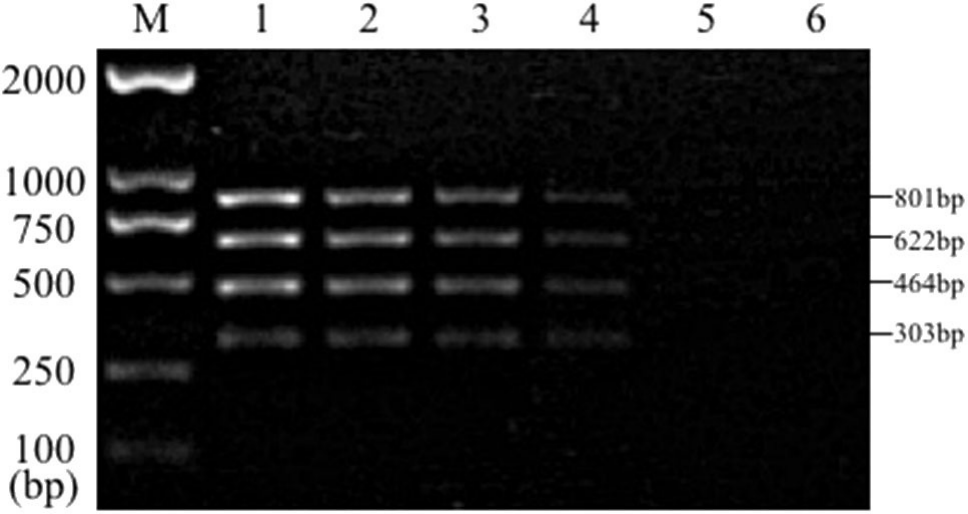
Results of multiplex PCR assay sensitivity experiment. Lane M, D2000 DNA marker; Lane 1, 100 ng of DNA for each of *Salmonella, E. coli, S. aureus*, and *K. pneumoniae*; Lane 2, 10 ng; Lane 3, 1 ng; Lane 4, 100 pg; Lane 5, 10 pg; Lane 6, 1 pg.

Sensitivity test results of the reference bacteria DNA demonstrated that, the single PCR assay was capable of properly identifying the presence of bacteria at the following lowest concentration, 1.0 pg/μL for *S. aureus*, 10.0 pg/μL for *E. coli*, Salmonella, and *K. pneumoniae* (Figure 3). The multiplex PCR assay could properly identify the presence of bacteria at 100 pg/μL of DNA template for *S. aureus, E. coli, Salmonella*, and *K. pneumoniae* (Figure 4).

### Results of Multiplex PCR Specificity Experiment

**Figure 5:**
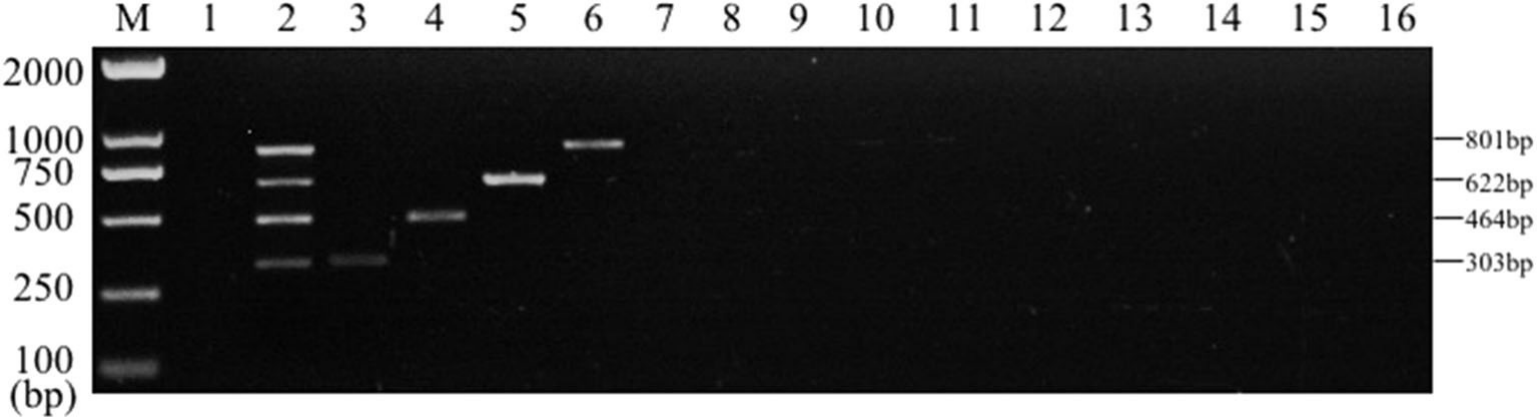
Results of multiplex PCR specificity experiment. Lane M, D2000 DNA marker; Lane 1, Negative control; Lane 2, mixed DNA template of *Salmonella, E. coli, S. aureus* and *K. pneumoniae*; Lane 3, *K. pneumoniae*; Lane 4, *S. aureus*; Lane 5, *E. coli*; Lane 6, *Salmonella*; Lane 7, *Clostridium perfringens*; Lane 8, *Pasteurella multocida*; Lane 9, *P. aeruginosa*; Lane 10, *Streptococcus pneumoniae*; Lane 11, *Proteus mirabilis*; Lane 12, *Staphylococcus sciuri*; Lane 13, *Staphylococcus pseudintermedius*; Lane 14, *Acinetobacter baumannii*; Lane 15, *Enterococcus faecalis*; Lane 16, *Bacillus subtilis*.

PCR specificity test results of bacteria DNA indicated that, the multiplex PCR assay could be capable to effectively identify the mixed DNA and the single DNA samples. Conversely, no bands of common clinical bacteria, including *Clostridium perfringens, P. aeruginosa, Pasteurella multocida, Streptococcus pneumoniae, Streptococcus pneumoniae, Proteus mirabilis, Staphylococcus sciuri, Staphylococcus pseudintermedius, Acinetobacter baumannii, Enterococcus faecalis*, and *Bacillus subtilis* were amplified by multiplex PCR (Figure 5). However, no amplification was achieved from control groups of other bacteria. The results suggested that the established multiplex PCR method showed good specificity.

### Results of Multiplex PCR Stability Experiment

**Figure 6:**
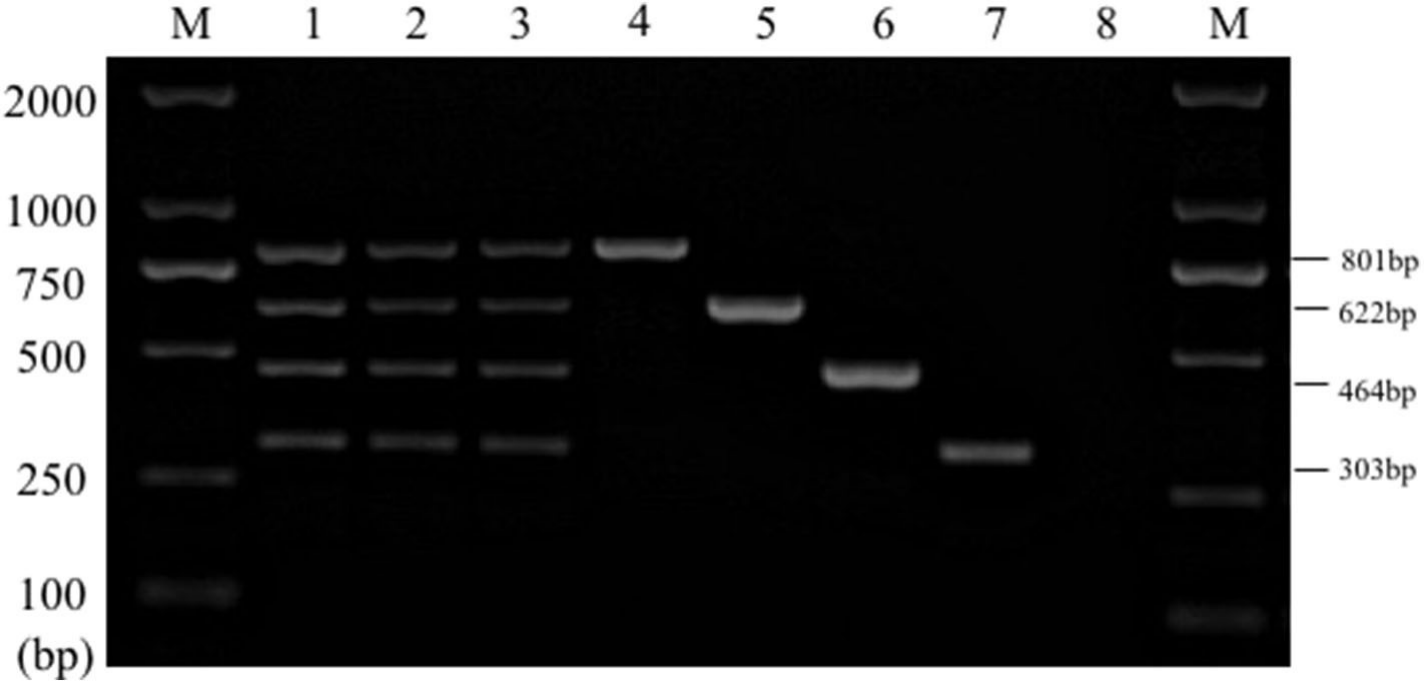
Results of multiplex PCR stability experiment. Lane M, D2000 DNA marker; Lane 1~3: mixed DNA template; Lane 4, *Salmonella*; Lane 5, *E. coli*; Lane 6, *S. aureus*; Lane 7, *K. pneumoniae*; Lane 8, Negative control.

The stability test results displayed that the specific target bands were found in all the positive samples, while no amplification products were found in the negative samples (Figure 6). The above results were in line with our expectations and the experimental requirements. Together these results suggested that the established multiplex PCR method had good stability and repeatability.

### Detection and Identification Results of Clinical Samples

Three hundred eighty samples were detected by multiplex and single PCR method established in current study. And the results were shown at Tables 2, 3. The results of the Table 2 demonstrated that among the 368 carcass samples, positive detection rates of *E. coli, K. pneumoniae, Salmonella*, and *S. aureus* were 33.7% (124/368), 12.0% (44/368), 10.6% (39/368), and 13.9% (51/368), respectively. Among the 12 visceral tissue samples, positive detection rates of *E. coli, K. pneumoniae, Salmonella*, and *S. aureus* were 41.7% (5/12), 25.0% (3/12), 16.7% (2/12), and 8.3% (1/12), respectively. Positive detection rates of multiplex PCR were consistent with that of single PCR (Table 2). At the same time, the results also showed that there were four kinds of bacteria contamination in mink carcasses.

**Table 2:** Multiplex and single PCR detection results of clinical samples from Shandong Province, China.

**Samples**	**Pathogens**	**Proportion of positive samples (%)**		**Concordance rate (%)**
		**Multiplex PCR**	**Single PCR**	
Carcass samples	*Salmonella*	39/368 (10.6)	39/368 (10.6)	100
	*E.coli*	124/368 (33.7)	124/368 (33.7)	100
	*K. pneumoniae*	44/368 (12.0)	44/368 (12.0)	100
	*S. aureus*	51/368 (13.9)	51/368 (13.9)	100
Visceral tissue	*Salmonella*	2/12 (16.7)	2/12 (16.7)	100
	*E. coli*	5/12 (41.7)	5/12 (41.7)	100
	*K. pneumoniae*	3/12 (25)	3/12 (25)	100
	*S. aureus*	1/12 (8.3)	1/12 (8.3)	100

**Table 3:** Co-contamination detection results of pathogenic bacteria in 380 clinical samples by multiplex PCR.

**Pathogens**	**Proportion of positive samples (%)**	
	**carcass samples (368)**	**visceral tissue (12)**
*E. coli* + *K. pneumoniae*	22 (6.0%)	1(8.3%)
*E. coli* + *Salmonella*	17 (4.6%)	1(8.3%)
*E. coli* + *S. aureus*	5 (1.4%)	0 (0)
*S. aureus* + *Salmonella*	5 (1.4%)	0 (0)
*E. coli* + *K. pneumoniae* + *Salmonella*	5 (1.4%)	0 (0)
*E. coli* + *Salmonella* + *S. aureus*	3 (0.8%)	0 (0)
*E. coli* + *K. pneumoniae* + *Salmonella* + *S. aureus*	3 (0.8%)	0 (0)
Total	60 (16.4%)	2 (16.7%)

Co-contamination detection results of bacteria in 380 clinical tissue samples by multiplex PCR were shown at Table 3. From the results of Table 3, it could be seen that single and multiple contamination were present in these samples. Among the 368 carcass samples, positive detection rates of dual contamination for *E. coli, K. pneumoniae, E. coli*, and *Salmonella* were 6.0% (22/368) and 4.6% (17/368), respectively. Positive detection rates of dual contamination for *E. coli, S. aureus, Salmonella*, and *S. aureus* were 1.4% (5/368). Positive detection rate of triple contamination for *E. coli, K. pneumoniae*, and *Salmonella* was 1.4% (5/368). Positive detection rate of triple contamination for *E. coli, Salmonella*, and *S. aureus* was 0.8% (3/368). Positive detection rate of quadruple contamination was 0.8% (3/368). Among the 12 visceral tissue samples, positive detection rates of dual contamination for *E. coli, K. pneumoniae, E. coli*, and *Salmonella* were 8.3% (1/12). Together these results suggest that different degrees of double, triple, or quadruple bacterial infection were present in the minks used for tissue sampling. The multiplex PCR could detect four kinds of bacteria from contaminated mink carcasses.

## Discussion

Specific primers were designed for specific target genes of four kinds of bacteria in the current study. The *nuc* gene of *S. aureus* encodes an extracellular thermostable nuclease, which is often used to detect *S. aureus* rapidly and specifically (15, 17, 22, 23). The *invA* gene of *Salmonella* is responsible for encoding the surface protein of the infected epithelial cells, which is common within the genus and unique among the genera, and is closely related to the pathogenicity of *Salmonella* (24, 25). When using *invA* gene as the target gene to design primers for *Salmonella* detection, strong specificity and detection accuracy can be obtained (26). Housekeeper gene *phoA* is used as a specific target gene for detection of *E*. *coli* (16, 27, 28). *khe* gene encodes the unique hemolysin of *K. pneumoniae* and is widely used in its detection (29, 30). Four pairs of specific primers designed in this study were used for the multiplex PCR, and the size intervals of the expected amplification products were more than 100 bp, so that different target genes could be distinguished after agarose gel electrophoresis.

The results of single PCR showed that the four pairs designed in this study could amplify the corresponding target genes specifically. Therefore, these primers can be used in multiplex PCR detection system. The optimization of reaction conditions is the key to the construction of multiplex PCR system, the most important of which is the optimization of annealing temperature. Generally, the annealing temperature is determined according to the chain breaking temperature of the upstream and downstream primers, but sometimes the results are not the same as expected (31). Although a single target gene fragment can be amplified specifically at 56–60°C, the annealing temperature of 4–6°C can be reduced in the multiplex PCR reaction, which is conducive to the amplification of all target gene fragments (32). The optimal annealing temperature is determined by designing the annealing temperature gradient, and the optimization of primer concentration, primer addition amount and addition proportion is also an important step of the optimization scheme (18). Our study showed that under the same cycle number, the amplification efficiency of specific primers for *E. coli* and *S. aureus* was higher than that for *Salmonella* and *K. pneumoniae*. The amplification efficiency of each pair of primers could be effectively balanced by reducing the concentration of primers with high amplification efficiency and increasing the concentration of primers with low amplification rate (33).

In this study, the sensitivity test results showed that the minimum detection amount of multiplex PCR for four pathogens reached 100 pg. The multiplex PCR sensitivity of *E. coli, Salmonella*, and *S. aureus* in this study is close to or higher than that reported by Xu et al. (34) and Wang et al. (16). The minimum detection concentration of single PCR for bacteria DNA could reach 10.0 pg/μL, even 1.0 pg/μL. The single PCR sensitivity of *E. coli* detection in the current study is the same as that of Xu et al. (34), and it is more convenient and time-saving than that of Guan et al. (35) and Liu et al. (36). In this study, 380 samples were detected by multiplex PCR and single PCR. The results showed that the positive detection rate, accuracy, and sensitivity of multiplex PCR were in agreement with that of single PCR. The multiplex PCR method established in this study can be used to identify and detect bacteria in mink tissue samples. This sensitivity can meet the needs of clinical detection. The specificity test results showed that the multiplex PCR system could not only amplify the mixed samples and single samples, but also could not amplify other kinds of common pathogens or opportunistic pathogens in minks which cause respiratory tract and digestive tract diseases. All of these show that the method is more specific and can be applied to the detection and identification of specific pathogenic bacteria. The research of Guan et al. (37) also showed good stability and repeatability of multiplex PCR, which was consistent with our results.

The detection results of clinical samples showed that the single and co-infection of bacteria in mink visceral tissue samples and carcass samples in Shandong are serious, which suggests that enough attention should be paid to these multiple and single infections. This is not consistent with the research results in pigs (35), which may be due to the different composition of sample pathogens caused by factors such as pre-mortem health status, feeding, and storage conditions.

## Conclusions

In conclusion, the multiplex PCR method is designed to detect and analyze the pathogenic microorganisms in mink carcass and viscera, which provides a rapid, specific and sensitive detection method for the identification of pathogenic bacteria in minks. The establishment of the multiplex PCR is conducive to the harmless treatment, development and utilization of mink carcass resources, and provides technical support for the safe and accurate application of fur animal carcass resources.

## Data Availability Statement

The original contributions presented in the study are included in the article/Supplementary Materials, further inquiries can be directed to the corresponding author/s.

## Ethics Statement

This study was carried out in strict accordance with the recommendations of the Animal Ethics Committee of Shandong Animal Protection and Welfare Institute (Number: SDAUA-2018-47). Moreover, samples collecting treatment and biosafety in this study were performed in accordance with national and local laws and guidelines.

## Author Contributions

HG conceived and designed the experiments. PL, DZ, and HL are mainly responsible for experimental implementation. JQ and JP are mainly responsible for sample collection and helped to do some experiments. PL and JQ wrote the manuscript. All authors have read and approved the final manuscript.

## Conflict of Interest

The authors declare that the research was conducted in the absence of any commercial or financial relationships that could be construed as a potential conflict of interest.
